# Reimagining a pass/fail clinical core clerkship: a US residency program director survey and meta-analysis

**DOI:** 10.1186/s12909-023-04770-8

**Published:** 2023-10-24

**Authors:** Andrew Wang, Krystal L. Karunungan, Jacob D. Story, Nathan A. Shlobin, Jiyun Woo, Edward L. Ha, Karen E. Hauer, Clarence H. Braddock

**Affiliations:** 1 David Geffen School of Medicine, University of California, Los Angeles, 10833 Le Conte Ave, Los Angeles, CA 90095 USA; 2 College of Medicine, Charles R. Drew University of Medicine and Science, Los Angeles, CA USA; 3 Feinberg School of Medicine, Northwestern University, Chicago, IL USA; 4 Crean Lutheran High School, Irvine, CA USA; 5 University of California, San Francisco School of Medicine, San Francisco, CA USA

**Keywords:** Medical education, Educational assessment, Clerkship, Residency and internship, Applicant selection

## Abstract

Pass/fail (P/F) grading has emerged as an alternative to tiered clerkship grading. Systematically evaluating existing literature and surveying program directors (PD) perspectives on these consequential changes can guide educators in addressing inequalities in academia and students aiming to improve their residency applications.

In our survey, a total of 1578 unique PD responses (63.1%) were obtained across 29 medical specialties. With the changes to United States Medical Licensure Examination (USMLE), responses showed increased importance of core clerkships with the implementation of Step 2CK cutoffs. PDs believed core clerkship performance was a reliable representation of an applicant’s preparedness for residency, particularly in Accreditation Council for Graduate Medical Education’s (ACGME)Medical Knowledge and Patient Care and Procedural Skills. PDs disagreed with P/F core clerkships because it more difficult to objectively compare applicants. No statistically significant differences in responses were found in PD preferential selection when comparing applicants from tiered and P/F core clerkship grading systems. If core clerkships adopted P/F scoring, PDs would further increase emphasis on narrative assessment, sub-internship evaluation, reference letters, academic awards, professional development and medical school prestige.

In the meta-analysis, of 6 studies from 2,118 participants, adjusted scaled scores with mean difference from an equal variance model from PDs showed residents from tiered clerkship grading systems overall performance, learning ability, work habits, personal evaluations, residency selection and educational evaluation were not statistically significantly different than from residents from P/F systems.

Overall, our dual study suggests that while PDs do not favor P/F core clerkships, PDs do not have a selection preference and do not report a difference in performance between applicants from P/F vs. tiered grading core clerkship systems, thus providing fertile grounds for institutions to examine the feasibility of adopting P/F grading for core clerkships.

**Supplementary Information:**

The online version contains supplementary material available at 10.1186/s12909-023-04770-8.

## Introduction

Assessment of student performance in core clinical clerkships leads to grade assignments which are associated with residency selection by program directors (PD). Pass/fail (P/F) grading has emerged as an alternative to tiered clerkship grading [1]. Proponents contend that P/F grading promotes the development of a foundation for self-regulated learning and reduces grade inflation while promoting student wellness and minimizing racial and ethnic disparities [2, 3]. However, others argue that P/F grading increases stress, removes objective measures that allow differentiation on residency applications. Nonetheless, P/F grading has been widely adopted for preclinical coursework and United States Medical Licensure Examination (USMLE) Step 1 to P/F in January 2022. Many medical schools have temporarily adopted P/F grading in response to the COVID-19 pandemic following the guidance of the Liaison Committee on Medical Education (LCME) [4]. These changes have spurred further discussions on the potential implications of permanently adopting a P/F core clerkship. Systematically evaluating existing literature and surveying PD perspectives on these consequential changes can guide educators in addressing inequalities in academia and students aiming to improve their residency applications.

## Methods

For the survey, the authors manually queried a subset (2500 of more than 5000 programs, outreach > 50% for every medical specialty except internal medicine and family medicine) of valid PD emails through the ACGME public 2021–2022 List of Specialty Programs (*n* = 29). In rounds (1/2021-12/2021), PDs were contacted. This was 7-item anonymous online survey using the ExpertReview validation tool (Qualtrics XM operating system version X4 [Qualtrics International Inc]). The survey (using Qualtrics and Google Forms) (Supplementary Table 6) included questions on PD demographics. PDs were then prompted for their general perceptions regarding the impact of P/F clerkships in the context of changes to Step 1 and Step 2 CS on residency preparedness, selection and institutional disparities. Responses were recorded on 3-point Likert scales (disagree, neutral, agree) and reported as counts and percentages. Derived 95% confidence intervals (CI) were defined by AAPOR guidelines (Supplementary Table 3). Statistically significance (*P* < 0 0.05) was considered by nonoverlapping 95% CI using Stata statistical software (StataCorp version 16.1). Subgroup analyses between regions and between AAMC–defined primary care (internal medicine, family medicine, pediatrics, internal medicine/pediatrics) and nonprimary care specialties were complete. Surveys with incomplete PD demographics were excluded (*n* = 11) and incomplete surveys (< 3%) were censored. This study was IRB exempt because it used deidentified data.

For the meta-analysis, Embase, PubMed, and Scopus was searched since inception through 01/01/2022 (Supplementary Table 1) with no restrictions. Studies exploring P/F clerkship grading in the context of a cohort of PD assessments were included. Reviewers assessed study characteristics, clinical and nonclinical resident performance with PD’s personal evaluation (worse:0 to best:100). This study followed the PRISMA guidelines (Supplementary Table 2).

## Results

The total survey response rate was 63.1% [*n* = 1578] (Table 1). The majority of participants were 50 ± 10 years old and male (63.0% [*n* = 994]); had served as program directors for an average of 6.8 ± 6.2 years and were distributed across US regions (Northeast 30.4% [*n* = 480], Midwest 25.2% [*n* = 398], South 24.0% [*n* = 378], West 20.4% [*n* = 322]). Family Medicine (13.1% [*n* = 204]), Internal Medicine (9.8% [*n* = 155]), Surgery (7.0% [*n* = 110]), were the most commonly represented specialties. More responses from non-primary care (72.4% [*n* = 1082]) specialties were collected than primary care specialties (31.4% [*n* = 496]). Since changes to USMLE Step 1 to P/F and Step 2CS being discontinued, currently many PDs will implement a Step 2 CK cutoff score (71.2%, CI, 68.1–74.3; *n* = 1124), but no cutoff’s in NBME score or minimum number of professional activities (research, community service, leadership) or supplemental application material would be required.

**Table 1:** Program director perspectives on residency preparedness and applicant selection following the change to pass/fail core clerkship grading

**A.**					
**Specialty**				**Respondents (N)**	**Response Rate (%)**
Anesthesiology				51/77	66.2
Child neurology				25/38	65.8
Dermatology				45/72	62.5
Emergency medicine				84/130	64.6
Family medicine				204/300	68
Internal medicine				155/229	68
Internal medicine/Pediatrics				61/108	56.5
Interventional radiology (integrated and independent)				26/45	57.8
Medical genetics and genomics				14/24	58.3
Neurologic surgery				36/59	61
Neurology				47/80	58.8
Nuclear medicine				7/19	36.8
Obstetrics and gynecology				91/132	68.9
Ophthalmology				41/63	65.1
Orthopedic surgery				63/101	62.4
Otolaryngology				38/62	61.3
Pathology				47/71	66.2
Pediatrics				76/106	71.7
Physical medicine and rehabilitation				24/47	51.1
Plastic surgery (integrated and independent)				35/52	67.3
Preventive medicine				17/36	47.2
Psychiatry				77/121	63.6
Radiation oncology				21/46	45.7
Radiology-diagnostic				57/97	58.8
Surgery				110/168	65.5
Thoracic surgery				21/37	57.8
Transitional year				37/81	45.7
Urology				50/73	68.5
Vascular surgery (integrated)				18/32	56.3
**B.**					
**ACGME Core Competencies**	**Professionalism and Ethics**	1551	25.70%	40.50%	33.80%
			(22.6-28.8)	(37.4-43.6)	(30.7-36.9)
	**Interpersonal and Communication Skills**	1554	24.70%	40.00%	35.30%
			(21.6-27.8)	(26.9-43.1)	(32.2-38.4)
	**Medical Knowledge**	1570	18.30%	28.30%	53.40%
			(15.2-21.4)	(25.2-31.4)	(50.3-56.5)
	**Systems-Based Practice**	1569	23.50%	43.50%	33.00%
			(20.4-26.6)	(40.4-46.6)	(29.9-36.1)
	**Patient Care and**	1569	21.10%	33.20%	45.70%
	**Procedural Skills**		(18.0-24.2)	(30.1-36.3)	(42.6-48.8)
	**Practice-Based Learning and Improvement**	1562	22.60%	39.90%	37.50%
			(19.5-25.7)	(36.8-44.0)	(34.4-40.6)
**Residency applicant selection factors**	**Clerkship narrative assessment**	1571	7.20%	14.40%	78.40%
			(4.1-10.3)	(11.3-17.5)	(74.3-81.5)
	**Step 2 CK**	1571	4.70%	12.10%	83.20%
			(1.6-7.8)	(8.0-15.2)	(80.1-86.3)
	**NBME scores**	1555	14.70%	41.70%	43.60%
			(11.6-17.8)	(38.6-44.8)	(40.5-46.7)
	**Medical school prestige**	1567	19.80%	27.50%	52.70%
			(16.7-32.9)	(24.4-30.6)	(49.6-55.8)
	**Reference letters**	1568	8.70%	25.40%	65.90%
			(5.6-11.8)	(22.3-28.5)	(62.8-69.0)
	**Sub-internship evaluation**	1570	4.00%	24.20%	71.80%
			(0.9-7.1)	(21.1-27.3)	(68.7-74.9)
	**Personal statement**	1559	12.40%	45.90%	41.70%
			(9.3-15.5)	(42.8-49.0)	(38.6-44.8)
	**Professional development activities**	1562	7.10%	34.40%	58.50%
			(4.0-10.2)	(31.1-37.5)	(55.4-61.6)
	**Academic awards or special honor societies**	1565	5.70%	26.30%	68.00%
			(2.6-8.8)	(23.2-29.4)	(64.9-71.1)
**Academic Inequalities**	**Medical student burnout**	1562	52.80%	33.50%	13.70%
			(49.7-55.9)	(30.4-36.6)	(10.6-16.8)
	**Gender and racial/ethnic disparities**	1529	55.10%	28.00%	16.90%
			(52.0-58.2)	(24.9-31.1)	(13.8-20.0)
	**Grade inflation**	1566	34.70%	21.20%	44.10%
			(31.6-37.8)	(18.1-24.3)	(41.0-47.2)
	**Variations in tiered grading distribution**	1565	32.70%	24.80%	42.50%
			(29.6-35.8)	(21.7-27.9)	(39.4-45.6)

PDs believed (81.9%; 95% CI, 78.8–85.0; *n* = 1292) core clerkship performance was a reliable representation of an applicant’s preparedness for residency, particularly in Medical Knowledge (53.4%; 95% CI, 50.3–56.5; *n* = 838) and Patient Care and Procedural Skills (45.7%; 95% CI, 42.6–48.8; *n* = 717) (Table 1). PDs disagreed with P/F core clerkships (88.9%; 95% CI, 85.8–92.0; *n* = 1403), expressed concerns that P/F core clerkships would make it more difficult to objectively compare residency applicants (96.4%; 95% CI, 93.3–99.5; *n* = 1521) and make the applicant screening more arduous (86.5%; 95% CI, 83.4–89.6; *n* = 1365). Yet, no statistically significant differences in responses were found in PD preferential selection when comparing applicants from tiered and P/F core clerkship grading systems. If core clerkships adopted P/F scoring, PDs would further increase emphasis on Step 2 CK performance (83.2%; 95% CI, 80.1–86.3; *n* = 1307), narrative assessment (78.4%; 95% CI, 74.3–81.5; *n* = 1232), sub-internship evaluation (71.8%; 95% CI, 68.7–74.9; *n* = 1127), reference letters (65.9%; 95% CI, 62.8–79.0; *n* = 1033), academic awards or special honor societies (68.0%, 95% CI, 64.9–71.1; *n* = 1064), professional development (58.5%; 95% CI 55.4–61.6; *n* = 914) and medical school prestige (52.7%; 95% CI, 51.1–57.3; *n* = 826). Findings for reference letters remained significant only among non-primary care PD specialties. Finally, in addressing academic inequalities in core clerkship, while PDs agreed changing core clerkship to P/F would help improve grade inflation (44.1%; 95% CI, 41.0-47.2; *n* = 691) and variations in tiered grading distributions (42.5%; 95% CI, 39.3–45.5; *n* = 665), PDs did not agree gender and racial/ethnic disparities (55.1%; 95% CI, 52.0-58.2; *n* = 842) and burnout (52.8%; 95% CI, 49.7–55.9; *n* = 825) would be improved.

 In the meta-analysis, 6 studies from 4,931 studies were identified with 2,118 participants at a median response rate of 81.0% (Supplementary Table 5) [5–10]. Overall, 7 specialties from PD respondents were represented and all studies were published before 2000 and were nonrandomized control trials (Supplementary Table 4). Reported as means, there was no difference in PD preference for residents from P/F or tiered grading system throughout residency training (37.0% Tiered; 95% CI, 0-100, *p* > 0.05). Adjusted scaled scores with mean difference from an equal variance model from PDs showed residents from tiered clerkship grading systems overall performance (5.5; 95% CI, 0.0-12.9), learning ability (2.7; 95% CI, 0.0-5.4), work habits (2.9; 95% CI, 0.0-5.8), personal evaluations (-1.6; 95% CI, -3.8-0.6) and educational evaluation (1.7; 95% CI, 0.0-4.3) were not statistically significantly different than from residents from P/F systems. However, there was a difference in the number of qualities of work products produced (6.8; 95% CI, 1.4–12.2, *p* < 0.0001). Meta-regression standard difference in means revealed no difference in tiered system residents’ overall performance in residency compared to P/F applicants (0.0001 fixed, *p* > 0.05; -0.0047 random, *p* > 0.015) (Table 2).

**Table 2:**
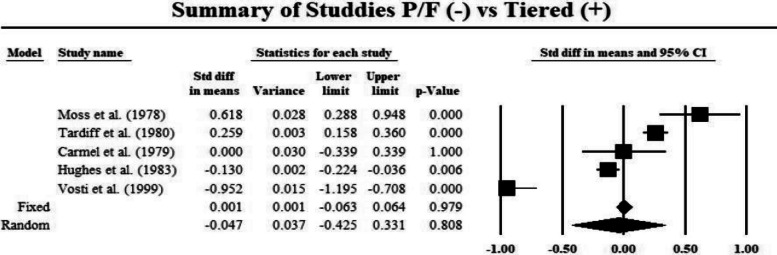
Forest Tree Plot of studies examining PD overall performance assessment between residents from tiered or P/F clerkship grading

## Discussion

The Coalition for Physician Accountability Review Committee has recommendations for changes to the residency match process – bringing a new paradigm that moves away from the “overreliance on licensure examination scores in the absence of valid, trustworthy measures of students’ competence and clinical abilities”. Our findings suggest that while PDs do not favor P/F core clerkships, PDs do not have a selection preference and do not report a difference in performance between applicants from P/F vs. tiered grading core clerkship systems.

The ACGME Outcomes Project Advisory Committee has established a framework of clinical competencies to guide medical schools in developing their clinical education programs. Perhaps as a result, PDs believed that core clerkship performance was a reliable representation of an applicant’s preparedness for residency. However, as ACGME continues to favor outcome-based measurements [11], medical schools are now expected to demonstrate how they use educational outcomes to improve student performance with little guidance. PDs did not feel strongly about whether the use of a tiered grading system for clerkship is adequate in ensuring that the ACGME clinical competencies are achieved. Shifting to P/F may allow institutions to focus on improving the quality of clerkship MSPE letters through greater emphasis on direct observation and real-time feedback [12].

The expansion of P/F grading in medical education - from preclinical coursework to Step 1 to core clerkships - has been driven by studies advocating for its potential to improve learning, wellness and academia inequalities [3]. Conversely, tiered clerkship grades and narrative assessments have been shown to be biased against underrepresented minority students, impeding efforts to improve diversity across specialties [2]. While PDs agreed that transitioning core clerkships to P/F would improve grade inflation and variations in tiered grading distributions, they did not believe racial, ethnic or gender disparities or burnout would improve. Further study is needed not only to balance calls for a P/F medical curriculum with the need for objective metrics, but also to determine whether doing so can sufficiently address existing disparities [13].

Several limitations of this study should be considered. First, the meta-analysis had a relatively small number of studies and medical specialties included, with all studies published prior to the year 2000 representing a different environment for resident selection compared to day. However, our prospective survey of PDs across specialties demonstrated similar results. Second, the meta-analysis’s resident survey assessment questions were not standardized and often normative perceptions, only quantitative data was summarized utilizing adjusted mean differences to compare performances. Third, while the survey total number of respondents was high, overall response rate across all specialties was insufficient to avoid selection and availability heuristic bias which limits generalizability. However, no difference was observed during subgroup and sensitivity analysis. Finally, this study focused on PDs associated with MD degree granting programs and may not be applicable to DO related programs.

We suggest that the COVID-19 pandemic has provided fertile grounds for institutions to examine the feasibility of adopting P/F grading for core clerkships. As educators begin to decide the extent to which their curricula will be shaped by the pandemic, medical education remains at a turning point.

### Supplementary Information

**Additional file 1: Supplementary Table 1.** Search Strategy. **Supplementary Table 2.** PRISMA Checklist. **Supplementary Table 3.** AAPOR Disclosure Checklist. **Supplementary Table 4.** Characteristics of the Included Studies Examining PD Perceptions on P/F Clerkship [1-6]. **Supplementary Table 5.** Flow Chart of Study Selection to Quantitively Evaluate PD Perceptions of Residents from Schools with Tiered Versus P/F Clerkship Grading. **Supplementary Table 6.** Program Director Online Survey.

## Data Availability

The datasets used and/or analyzed during the current study are available from the corresponding author upon reasonable request.

## Abbreviations

AAEE: American Medical Education in Europe Guide
AAMC: Association of American Medical Colleges
ACGME: Accreditation Council for Graduate Medical Education
AAPOR: American Association for Public Opinion Research
CI: Confidence Interval
CK: Clinical Knowledge
CS: Clinical Skills
ERAS: Electronic Residency Application Service
FSMB: Federation of State Medical Boards
IRB: Institutional Review Board
LCME: Liaison Committee on Medical Education
MSPE: Medical Student Performance Evaluation
NBME: National Board of Medical Examiners
P/F: Pass/Fail
PRISMA: Preferred Reporting Items for Systematic Reviews and Meta-analyses
PD: Program Director
USMLE: United States Medical Licensure Examination
