# Supplementary material for: Reimagining a pass/fail clinical core clerkship: a US residency program director survey and meta-analysis
Source: BMC Med Educ. 2023 Oct 24;23:788. doi: 10.1186/s12909-023-04770-8 (PMC10598945; doi:10.1186/s12909-023-04770-8)
Supplement: Supplementary file 1 — Additional file 1: Supplementary Table 1. Search Strategy. Supplementary Table 2. PRISMA Checklist. Supplementary Table 3. AAPOR Disclosure Checklist. Supplementary Table 4. Characteristics of the Included Studies Examining PD Perceptions on P/F Clerkship [1-6]. Supplementary Table 5. Flow Chart of Study Selection to Quantitively Evaluate PD Perceptions of Residents from Schools with Tiered Versus P/F Clerkship Grading. Supplementary Table 6. Program Director Online Survey. [file 12909_2023_4770_MOESM1_ESM.docx]

**SUPPLEMENTARY**

**Supplementary Table 1.** Search Strategy

| **Search Engine** | **Search Terms** |
| --- | --- |
| PubMed | (“schools, medical”[MeSH] OR “education, medical, undergraduate”[MeSH] OR “medical school*”[tiab] OR “medical education*”[tiab] OR “medical curricu*”[tiab] OR “program director”[tiab] OR “residenc*”[tiab] OR “AAMC”[tiab] OR “american association of medical college”[tiab] OR “ACGME”[tiab] OR “Accreditation Council for Graduate Medical Education”[tiab] OR “LCME”[tiab] OR “Liaison Committee on Medical Education” OR “AAMC”[tiab] OR “american association of medical colleges”[tiab] OR “AMA”[tiab] OR “american medical association”[tiab] OR “FSMB” OR “federation of state medical boards”[tiab] OR “NBME”[tiab] OR “national board of medical exam*”[tiab] OR "undergraduate medical education"[tiab] OR “medical student education”[tiab]) AND (“clinical competence”[MeSH] OR “medical school grad*”[tiab] OR “pass/fail”[tiab] OR “pass-fail”[tiab] OR “p/f”[tiab] OR “traditional grad*”[tiab] OR “letter grad*”[tiab] OR “tier grad*”[tiab] OR “tiered grade”[tiab] OR “number grad*”[tiab] OR “hybrid grad*”[tiab] OR hierarch*[tiab] OR “grading scale*”[tiab] OR “grading system*”[tiab] OR “grading change*”[tiab] OR “scoring change*”[tiab] OR “preclinical grad*”[tiab] OR “clinical grad*”[tiab]) AND((“pass/fail”[tiab] OR “pass-fail”[tiab] OR “p/f”[tiab] OR “minimum passing standard*”[tiab]) OR (“preclinical curricul*”[tiab] OR “preclinical education”[tiab]) OR (“clinical clerkship”[MeSH] OR “clerkship*”[tiab] OR “clinical rotation”[tiab] OR “away rotation*”[tiab] OR “sub-intern*”[tiab] OR “subintern*”[tiab] OR “shelf exam*”[tiab] OR “NBME exam”[tiab]) OR (“residency match”[tiab] OR “NRMP”[tiab] OR “national resident matching program”[tiab] OR “ERAS”[tiab] OR “electronic residency application service*”[tiab] OR “residency placement*”[tiab] OR “applicant rank*”[tiab] or “applicant rating*”[tiab] or “application rank**”[tiab] or “application rating*”[tiab] OR “applicant selection*”[tiab] OR “selection criteri*”[tiab])) |
| Embase | ((“medical school*” OR “medical education*” OR “medical curricu*” OR “program director” OR “residenc*” OR “AAMC” OR “american association of medical college” OR “ACGME” OR “Accreditation Council for Graduate Medical Education” OR “LCME” OR “Liaison Committee on Medical Education” OR “AAMC” OR “american association of medical colleges” OR “AMA” OR “american medical association” OR “FSMB” OR “federation of state medical boards” OR “NBME” OR “national board of medical exam*” OR "undergraduate medical education" OR “medical student education”) AND (“clinical competence” OR “medical school grad*” OR “pass/fail” OR “pass-fail” OR “p/f” OR “traditional grad*” OR “letter grad*” OR “tier grad*” OR “tiered grade” OR “number grad*” OR “hybrid grad*” OR “hierarch*” OR “grading scale*” OR “grading system*” OR “grading change*” OR “scoring change*” OR “preclinical grad*” OR “clinical grad*”) AND ((“pass/fail” OR “pass-fail” OR “p/f” OR “minimum passing standard*”) OR (“preclinical curricul*” OR “preclinical education”) OR (“clinical clerkship” OR “clerkship*” OR “clinical rotation” OR “away rotation*” OR “sub-intern*” OR “subintern*” OR “shelf exam*” OR “NBME exam”) OR (“residency match” OR “NRMP” OR “national resident matching program” OR “ERAS” OR “electronic residency application service*” OR “residency placement*” OR “applicant rank*” or “applicant rating*” or “application rank*” or “application rating*” OR “applicant selection*” OR “selection criteri*”))):ti,ab,kw |
| Scopus | TITLE-ABS-KEY ( ( "medical school*" OR "medical education*" OR "medical curricu*" OR "program director" OR "residenc*" OR "AAMC" OR "american association of medical college" OR "ACGME" OR "Accreditation Council for Graduate Medical Education" OR "LCME" OR "Liaison Committee on Medical Education" OR "AAMC" OR "american association of medical colleges" OR "AMA" OR "american medical association" OR "FSMB" OR "federation of state medical boards" OR "NBME" OR "national board of medical exam*" OR "undergraduate medical education" OR "medical student education" ) AND ( "clinical competence" OR "medical school grad*" OR "pass/fail" OR "pass-fail" OR "p/f" OR "traditional grad*" OR "letter grad*" OR "tier grad*" OR "tiered grade" OR "number grad*" OR "hybrid grad*" OR "hierarch*" OR "grading scale*" OR "grading system*" OR "grading change*" OR "scoring change*" OR "preclinical grad*" OR "clinical grad*" ) AND ( ( "pass/fail" OR "pass-fail" OR "p/f" OR "minimum passing standard*" ) OR ( "preclinical curricul*" OR "preclinical education" ) OR ( "clinical clerkship" OR "clerkship*" OR "clinical rotation" OR "away rotation*" OR "sub-intern*" OR "subintern*" OR "shelf exam*" OR "NBME exam" ) OR ( "residency match" OR "NRMP" OR "national resident matching program" OR "ERAS" OR "electronic residency application service*" OR "residency placement*" OR "applicant rank*" OR "applicant rating*" OR "application rank*" OR "application rating*" OR "applicant selection*" OR "selection criteri*" ) ) ) |

**Supplementary Table 2.** PRISMA Checklist

| **Section and Topic** | **Item #** | **Checklist item** | **Location where item is reported** |
| --- | --- | --- | --- |
| **TITLE** | | |  |
| Title | 1 | Identify the report as a systematic review. | 1 |
| **ABSTRACT** | | |  |
| Abstract | 2 | See the PRISMA 2020 for Abstracts checklist. | 3 |
| **INTRODUCTION** | | |  |
| Rationale | 3 | Describe the rationale for the review in the context of existing knowledge. | 2 |
| Objectives | 4 | Provide an explicit statement of the objective(s) or question(s) the review addresses. | 2 |
| **METHODS** | | |  |
| Eligibility criteria | 5 | Specify the inclusion and exclusion criteria for the review and how studies were grouped for the syntheses. | 3 |
| Information sources | 6 | Specify all databases, registers, websites, organisations, reference lists and other sources searched or consulted to identify studies. Specify the date when each source was last searched or consulted. | 3 |
| Search strategy | 7 | Present the full search strategies for all databases, registers and websites, including any filters and limits used. | 3 |
| Selection process | 8 | Specify the methods used to decide whether a study met the inclusion criteria of the review, including how many reviewers screened each record and each report retrieved, whether they worked independently, and if applicable, details of automation tools used in the process. | 3 |
| Data collection process | 9 | Specify the methods used to collect data from reports, including how many reviewers collected data from each report, whether they worked independently, any processes for obtaining or confirming data from study investigators, and if applicable, details of automation tools used in the process. | 3 |
| Data items | 10a | List and define all outcomes for which data were sought. Specify whether all results that were compatible with each outcome domain in each study were sought (e.g. for all measures, time points, analyses), and if not, the methods used to decide which results to collect. | 3 |
|  | 10b | List and define all other variables for which data were sought (e.g. participant and intervention characteristics, funding sources). Describe any assumptions made about any missing or unclear information. | 1, 3 |
| Study risk of bias assessment | 11 | Specify the methods used to assess risk of bias in the included studies, including details of the tool(s) used, how many reviewers assessed each study and whether they worked independently, and if applicable, details of automation tools used in the process. | 3 |
| Effect measures | 12 | Specify for each outcome the effect measure(s) (e.g. risk ratio, mean difference) used in the synthesis or presentation of results. | 3 |
| Synthesis methods | 13a | Describe the processes used to decide which studies were eligible for each synthesis (e.g. tabulating the study intervention characteristics and comparing against the planned groups for each synthesis (item #5)). | 3 |
|  | 13b | Describe any methods required to prepare the data for presentation or synthesis, such as handling of missing summary statistics, or data conversions. | 3 |
|  | 13c | Describe any methods used to tabulate or visually display results of individual studies and syntheses. | 3 |
|  | 13d | Describe any methods used to synthesize results and provide a rationale for the choice(s). If meta-analysis was performed, describe the model(s), method(s) to identify the presence and extent of statistical heterogeneity, and software package(s) used. | 3 |
|  | 13e | Describe any methods used to explore possible causes of heterogeneity among study results (e.g. subgroup analysis, meta-regression). | 3 |
|  | 13f | Describe any sensitivity analyses conducted to assess robustness of the synthesized results. | 3 |
| Reporting bias assessment | 14 | Describe any methods used to assess risk of bias due to missing results in a synthesis (arising from reporting biases). | 3 |
| Certainty assessment | 15 | Describe any methods used to assess certainty (or confidence) in the body of evidence for an outcome. | 3 |
| **RESULTS** | | |  |
| Study selection | 16a | Describe the results of the search and selection process, from the number of records identified in the search to the number of studies included in the review, ideally using a flow diagram. | 4 |
|  | 16b | Cite studies that might appear to meet the inclusion criteria, but which were excluded, and explain why they were excluded. | 4 |
| Study characteristics | 17 | Cite each included study and present its characteristics. | 4 |
| Risk of bias in studies | 18 | Present assessments of risk of bias for each included study. | 4 |
| Results of individual studies | 19 | For all outcomes, present, for each study: (a) summary statistics for each group (where appropriate) and (b) an effect estimate and its precision (e.g. confidence/credible interval), ideally using structured tables or plots. | 4 |
| Results of syntheses | 20a | For each synthesis, briefly summarise the characteristics and risk of bias among contributing studies. | 4 |
|  | 20b | Present results of all statistical syntheses conducted. If meta-analysis was done, present for each the summary estimate and its precision (e.g. confidence/credible interval) and measures of statistical heterogeneity. If comparing groups, describe the direction of the effect. | 4 |
|  | 20c | Present results of all investigations of possible causes of heterogeneity among study results. | 4 |
|  | 20d | Present results of all sensitivity analyses conducted to assess the robustness of the synthesized results. | 4 |
| Reporting biases | 21 | Present assessments of risk of bias due to missing results (arising from reporting biases) for each synthesis assessed. | 4 |
| Certainty of evidence | 22 | Present assessments of certainty (or confidence) in the body of evidence for each outcome assessed. | 4 |
| **DISCUSSION** | | |  |
| Discussion | 23a | Provide a general interpretation of the results in the context of other evidence. | 5 |
|  | 23b | Discuss any limitations of the evidence included in the review. | 5 |
|  | 23c | Discuss any limitations of the review processes used. | 5 |
|  | 23d | Discuss implications of the results for practice, policy, and future research. | 5 |
| **OTHER INFORMATION** | | |  |
| Registration and protocol | 24a | Provide registration information for the review, including register name and registration number, or state that the review was not registered. | 3 |
|  | 24b | Indicate where the review protocol can be accessed, or state that a protocol was not prepared. | 3 |
|  | 24c | Describe and explain any amendments to information provided at registration or in the protocol. | 3 |
| Support | 25 | Describe sources of financial or non-financial support for the review, and the role of the funders or sponsors in the review. | 1 |
| Competing interests | 26 | Declare any competing interests of review authors. | 1 |
| Availability of data, code and other materials | 27 | Report which of the following are publicly available and where they can be found: template data collection forms; data extracted from included studies; data used for all analyses; analytic code; any other materials used in the review. | 1, 3 |

**Supplementary Table 3.** AAPOR Disclosure Checklist

| **BASIC DISCLOSURE ELEMENTS** | **DETAILS** |
| --- | --- |
| Survey Sponsor | UCLA |
| Survey/Data collection supplier | Qualtrics/Google Form |
| Population represented | Program Director listed ACGME Specialty |
| Sample Size | 2500 Valid Emails |
| Mode of data collection | Anonymous Online Survey |
| Type of sample | Probability, for individual emails. Queried outreach >50%+ for every medical specialty except internal medicine and family medicine. |
| Start and end dates of data collection | 1/2021-12/2021 |
| Margin of sampling error for total sample | +/- 3.1 percentage points at the 95% confidence level |
| Margin of sampling error for key subgroups | +/- 4.3 percentage points at the 95% confidence level |
| Are the data weighted? | No, however, primary care vs non primary care and regional differences subgroup analysis were performed |
| Is the data censored? | Yes, to individual response question |
| Contact for more information | Andrew Wang David Geffen School of Medicine andrewwang@mednet.ucla.edu |

**Supplementary Table 4.** Characteristics of the Included Studies Examining PD Perceptions on P/F Clerkship [1-6]


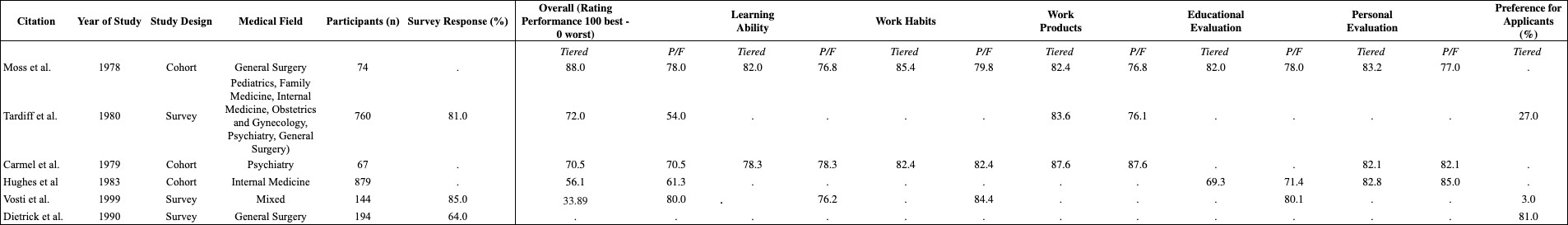


**Supplementary Table 5.** Flow Chart of Study Selection to Quantitively Evaluate PD Perceptions of Residents from Schools with Tiered
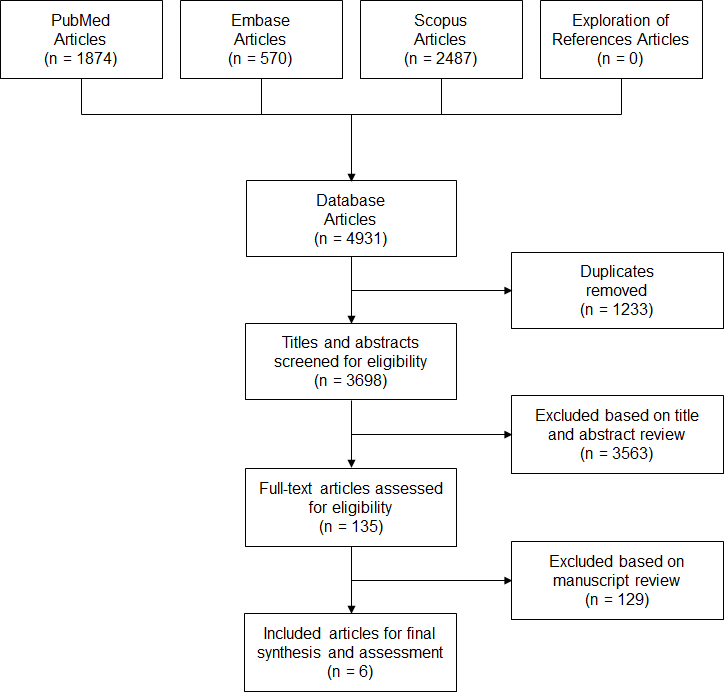
Versus P/F Clerkship Grading

**REFERENCES**

**Supplementary Table 6.** Program Director Online Survey

**Q1 Please complete the following demographics.**

**Q2 What is your current age?**

**________________________________________________________________**

**Q3 What is your current gender identity?**

**o Male (1)**

**o Female (2)**

**o Nonbinary or gender fluid (3)**

**Q4 How many years have you served as Residency Program Director at your current institution?**

**________________________________________________________________**

**Q5 In what region is your current residency program located in?**

**o Northeast (1)**

**o South (2)**

**o Midwest (3)**

**o West (4)**

| **Page Break** |  |
| --- | --- |

**Q6 Performance on core clerkships serves as a(n):**

|  | **Strongly Disagree (1)** | **Disagree (2)** | **Neutral (3)** | **Agree (4)** | **Strongly Agree (5)** |
| --- | --- | --- | --- | --- | --- |
| **Reliable representation of an applicant's preparedness for residency (1)** | **o** | **o** | **o** | **o** | **o** |
| **Objective measurement by which to screen and compare applicants (2)** | **o** | **o** | **o** | **o** | **o** |

**Q7 An applicant who received a “Pass” or higher in a tiered clerkship is equally as prepared for residency as one who received a “Pass” in a pass/fail clerkship.**

**o Disagree (1)**

**o Neutral (2)**

**o Agree (3)**

**Q8 When evaluating an applicant’s performance in core clerkships, I currently place greater emphasis on the narrative assessment than on the overall tiered clerkship grade, barring any failures or demerits.**

**o Disagree (1)**

**o Neutral (2)**

**o Agree (3)**

**Q9 Would you still increase emphasis on overall clerkship grades if pass/fail was used?**

**o Yes (1)**

**o No (2)**

| **Page Break** |  |
| --- | --- |

**Q10 Changing core clerkships to pass/fail:**

|  | **Strongly Disagree (1)** | **Disagree (2)** | **Neutral (3)** | **Agree (4)** | **Strongly Agree (5)** |
| --- | --- | --- | --- | --- | --- |
| **Will overall improve medical student preparedness for residency (1)** | **o** | **o** | **o** | **o** | **o** |
| **Will make it more difficult to objectively compare residency applicants (2)** | **o** | **o** | **o** | **o** | **o** |
| **Will make applicant screening more arduous (3)** | **o** | **o** | **o** | **o** | **o** |

**Q11 Changing core clinical clerkships to pass/fail will negatively impact a medical student’s ability to achieve the six ACGME Core Competencies for residency preparedness:**

|  | **Strongly Disagree (1)** | **Disagree (2)** | **Neutral (3)** | **Agree (4)** | **Strongly Agree (5)** |
| --- | --- | --- | --- | --- | --- |
| **Practice-Based Learning and Improvement (1)** | **o** | **o** | **o** | **o** | **o** |
| **Patient Care and Procedural Skills (2)** | **o** | **o** | **o** | **o** | **o** |
| **Systems-Based Practice (3)** | **o** | **o** | **o** | **o** | **o** |
| **Medical Knowledge (4)** | **o** | **o** | **o** | **o** | **o** |
| **Interpersonal and Communication Skills (5)** | **o** | **o** | **o** | **o** | **o** |
| **Professionalism and Ethics (6)** | **o** | **o** | **o** | **o** | **o** |

**Q12 I believe that changing core clerkships to pass/fail would help resolve the following issues:**

|  | **Disagree (1)** | **Neutral (2)** | **Agree (3)** |
| --- | --- | --- | --- |
| **Gender and racial/ethnic disparities (1)** | **o** | **o** | **o** |
| **Grade inflation (2)** | **o** | **o** | **o** |
| **Variations in tiered grading distribution (3)** | **o** | **o** | **o** |
| **Medical student burnout (4)** | **o** | **o** | **o** |

**-------------------------------------------------------------------------------------------------------------------------------**
